# Complete mitochondrial genome of yellow-rumped honeyguide *Indicator xanthonotus* (Piciformes: Indicatoridae)

**DOI:** 10.1080/23802359.2018.1532837

**Published:** 2018-10-26

**Authors:** Yubao Duan, Yuan Li, Dan Liang, Shimiao Shao, Xu Luo

**Affiliations:** a Key Laboratory for Forest Resources Conservation and Utilization in the Southwest Mountains of China, Ministry of Education, Southwest Forestry University, Kunming, Yunnan Province, China;; b College of Biodiversity Conservation and Utilization, Southwest Forestry University, Kunming, Yunnan Province, China

**Keywords:** *Indicator xanthonotus*, mitogenome, phylogeny

## Abstract

In this study, we first sequenced and described the complete mitochondrial genome and phylogeny of *Indicator xanthonotus*. The whole genome of *I. xanthonotus* was 17,603 bp in length and contained 14 protein-coding genes, 22 transfer RNA genes, two ribosome RNA genes, and two non-coding control regions. The overall base composition of the mitochondrial DNA was 31.09% for A, 27.34% for T, 28.96% for C, and 12.61% for G, with a GC content of 41.57%. A phylogenetic tree strongly supported that *I. xanthonotus* clustered with another Piciformes species by high probability.

The Yellow-rumped Honeyguide (*I. xanthonotus* Blyth, 1842) is distributed in southern and southeastern of Asia (China, Nepal, Myanmar, et al.) and inhabits in rocky gorges and valleys with broadleaved or coniferous forest between 1450 and 3500 m (BirdLife International 2018; Gill and Donsker 2018). This species is a brood parasite avian and was usually considered to have strong association with Giant honey bee *Apis dorsata* (Fleischer 2011; BirdLife International 2018). This species is listed as Near Threatened (NT) on the IUCN Red List (IUCN, 2018). Webb and Moore (2005) constructed a phylogenetic tree that analyzed intergeneric relationships combined 12S, Cyt b, and COI sequences. *I. xanthonotus*, as a scarce and poorly known species, very few studies had been reported, including complete mitochondrial genome not been revealed. Therefore, we sequenced the complete mitochondrial genome of *I. xanthonotus* to enhance our understanding on the phylogeny of Indicatoridae.

The specimen was collected from Mt. Gaoligong in Lushui County, which was located northwestern of Yunnan Province in China, and stored at College of Biodiversity Conservation and Utilization, Southwest Forestry University. The total mitochondrial DNA was extracted from the muscle tissue and sequenced using next-generation sequencing. The complete mitochondrial genome of *I. xanthonotus* was submitted to the NCBI database under the accession number MH737741. Phylogenetic tree of the relationships among Piciformes and Coraciiformes were presented using 16 species by maximum parsimony analyses using PAUP* version 4.0b10 software with 1000 bootstrap replication (Swofford 2002). Bayesian inference was calculated with MrBayes3.1.2 with a general time reversible (GTR) model of DNA substitution and a gamma distribution rate variation across sites (Ronquist and Huelsenbeck 2003). Sequences of Coraciiformes (*Eurystomus orientalis*) obtained from GenBank (NC_011716.2) were used as outgroups to root trees following Mccormack et al. (2013) and Eo (2017).

The complete mitochondrial genome of *I. xanthonotus* was 17,603 bp in length. A total of 40 mitochondrial genes were identified, including 14 protein-coding genes (PCGs), 22 transfer RNA (tRNA) genes, two ribosomal RNA (rRNA) genes, and two non-coding control regions (D-loop). Among these genes, ND6 and eight tRNAs (*tRNA^Gln^*, *tRNA^Ala^*, *tRNA^Asn^*, *tRNA^Cys^*, *tRNA^Tyr^*, *tRNA^Ser^*, *tRNA^Pro^*, and *tRNA^Glu^*) were located on the light strand (Lstrand), while all of the remaining genes were located on the heavy strand (H-strand). The overall base composition of *A. insignis* mitogenome was 31.09% for A, 12.61% for G 28.96% for C, and 27.34% for T, A + T content is 58.43%, which is higher than G + C content of 41.57%, similar to other Piciformes (Fuchs et al. 2015; Zhang et al. 2016; Eo 2017).

The reconstructed phylogenetic tree supported the placement of *I. xanthonotus* in Piciformes (Figure 1). In our results, 16 species were clustered into two groups*. Indicator xanthonotus* clustered with other Piciformes species and was strongly supported by the analyses of protein-coding genes. Thus, the mitochondrial genome reported here would be useful in the current understanding of the phylogeny and evolution of Piciformes.

**Figure 1.**
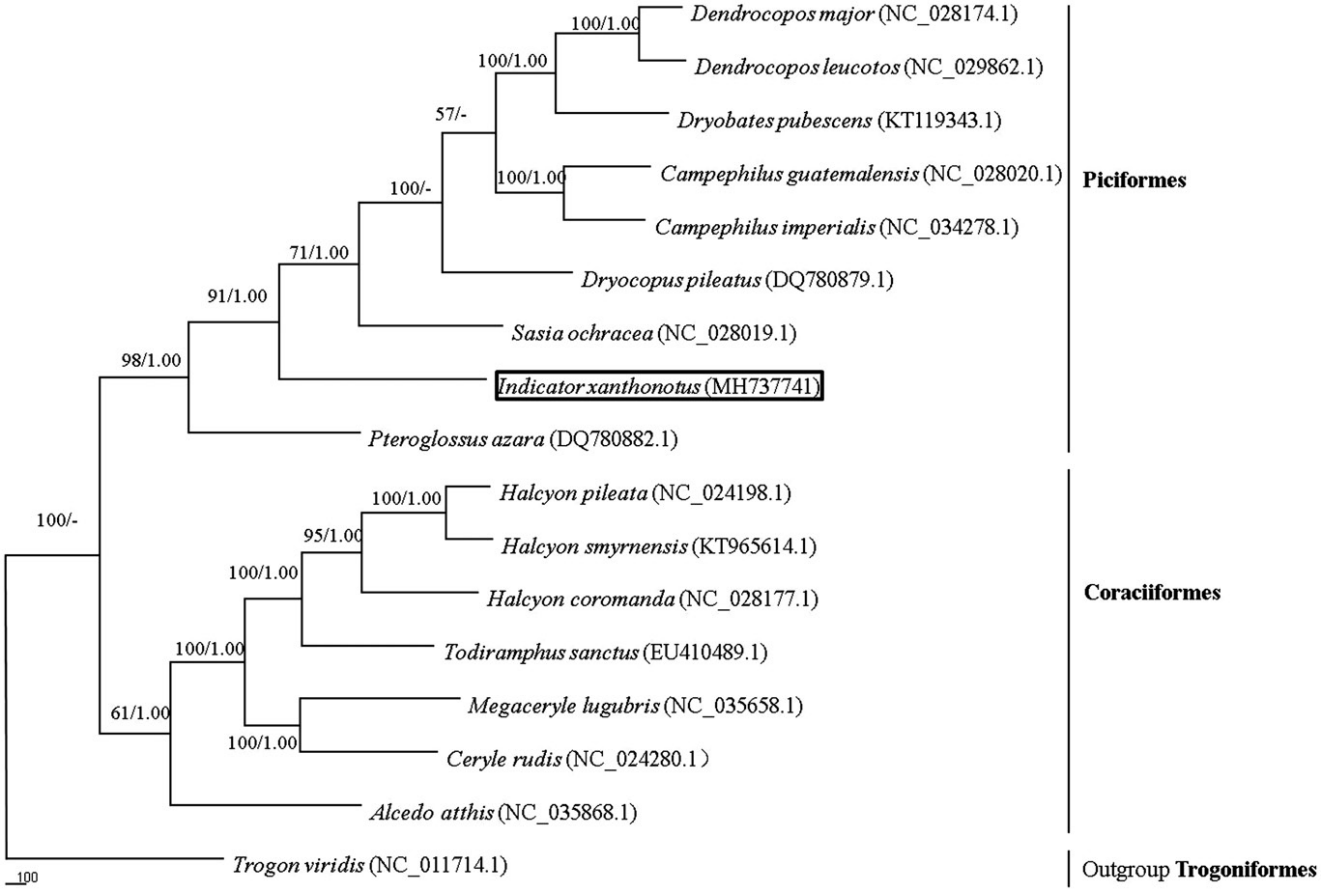
The phylogenetic tree based on combing protein-coding gene sequences of nine Piciformes and seven Coraciiformes. Numbers at a node of the tree branches represent maximum parsimony (BP, left) and Bayesian posterior probability (BPP, right).
